# Characteristics and Effectiveness of Co-Designed Mental Health Interventions in Primary Care for People Experiencing Homelessness: A Systematic Review

**DOI:** 10.3390/ijerph20010892

**Published:** 2023-01-03

**Authors:** Tobias Schiffler, Ali Kapan, Alina Gansterer, Thomas Pass, Lisa Lehner, Alejandro Gil-Salmeron, Daragh T. McDermott, Igor Grabovac

**Affiliations:** 1 Center for Public Health, Department of Social and Preventive Medicine, Medical University of Vienna, Kinderspitalgasse 15, 1090 Vienna, Austria; 2 Department of Science & Technology Studies, Cornell University, 303 Morrill Hall, Ithaca, NY 14853, USA; 3 International Foundation for Integrated Care, Wolfson College, Linton Rd., Oxford OX2 6UD, UK; 4 NTU Psychology, School of Social Sciences, Nottingham Trent University, Nottingham NG1 4FQ, UK

**Keywords:** homelessness, mental health, primary care

## Abstract

People experiencing homelessness (PEH) face a disproportionately high prevalence of adverse mental health outcomes compared with the non-homeless population and are known to utilize primary healthcare services less frequently while seeking help in emergency care facilities. Given that primary health services are more efficient and cost-saving, services with a focus on mental health that are co-designed with the participation of users can tackle this problem. Hence, we aimed to synthesize the current evidence of such interventions to assess and summarize the characteristics and effectiveness of co-designed primary mental healthcare services geared towards adult PEH. Out of a total of 10,428 identified records, four articles were found to be eligible to be included in this review. Our findings show that co-designed interventions positively impacted PEH’s mental health and housing situation or reduced hospital and emergency department admissions and increased primary care utilization. Therefore, co-designed mental health interventions appear a promising way of providing PEH with continued access to primary mental healthcare. However, as co-designed mental health interventions for PEH can improve overall mental health, quality of life, housing, and acute service utilization, more research is needed.

## 1. Introduction

Homelessness is strongly associated with significant health inequalities, including high levels of mortality and morbidity (e.g., cancer is the second most common cause of death) compared with the non-homeless population [1,2,3]. This association is observed in both directions, meaning homelessness in itself increases the risk of mental health problems with many people experiencing homelessness (PEH) disproportionately facing severe mental illness, cognitive impairment, as well as high rates of homicide and suicide [4,5,6]. International studies report a high prevalence of mental illness, such as depressive and anxiety disorder, among PEH ranging from 25% to 92%, exceeding the prevalence among people not experiencing homelessness [7,8,9]. According to a recently published systematic review by Gutwinski et al. [8] in 2021 surveying the prevalence of mental disorders among PEH in high-income countries, the most common diagnostic categories included alcohol use disorders, at 36.7% (95% CI 27.7 to 46.2) and drug use disorders, at 21.7% (95% CI 13.1 to 31.7), followed by schizophrenia spectrum disorders (12.4% [95% CI 9.5 to 15.7]) and major depression (12.6% [95% CI 8.0 to 18.2]). As this population continues to grow, the mental health of PEH is fast becoming a social and public health problem of great global importance [6].

Despite facing significant health challenges, PEH represent one of the most marginalized populations with their needs for mental healthcare being widely unmet [10]. Primary care provides the first point of contact for many people and also acts as a gatekeeper to other statutory services [11]. Yet, PEH are less likely to seek support and treatment through primary healthcare [2,12]. The reasons for not accessing primary care can be complex and interrelated [6], including economic issues (affordability), social stigma (prior rejection by health or social services), and organizational barriers (institutional schedules, complexity of the care system) [13,14]. In a study led by Farnarier et al. [15], PEH asked to complete a questionnaire listed “visiting a primary care physician” as only fifth on their list of priorities when they become ill. In turn, PEH’s healthcare pathways are characterized by high rates of visits to emergency departments and psychiatric hospitals for acute mental health needs [16,17]. The evidence for this is largely extended, including healthcare systems based and not based on the principle of universal healthcare coverage [6].

As it is an important opportunity to address or resolve health disparities when accessing primary healthcare, the question arises of what type of health intervention and support yields the best results. Although re-designing the current health and care systems to make primary healthcare services more accessible to PEH may result in several benefits, such as better health outcomes and integration into the community [8,18], it may present different challenges. A possible solution to this can be the implementation of co-designed healthcare models. Originating from the field of implementation science, the co-design approach is a specialized concept used in the development of target group-specific and person-centered health and social care models [19,20,21,22,23]. As co-designing methods pursue a holistic approach, including different groups of persons in the developmental process, stakeholders, researchers, and end users are provided the same opportunity to engage in the model-designing process and contribute their thoughts and ideas [24,25].

The unique aspect of this process results in the practicability of a model, as it is precisely adapted to real-life conditions and scenarios. Given such targeted interventions, resources of a financial or a temporal nature are used more cost-effectively and reduce the misalignment between researcher, stakeholder, and user aims [20]. Especially concerning underserved and marginalized groups of persons, the co-design approach is an invaluable tool to provide these individuals with the possibility of co-development and participation in decision-making processes [26,27]. Some findings from a cross-sectional study by Joyce and Limbos [28] and a quasi-experimental study by Stergiopoulos et al. [29] suggest that if collaborative primary healthcare models are not just tailored for PEH but also based in the community (i.e., located directly in homeless shelters or day centers), they may also be more effective in the identification, treatment, compliance, and improvement of mental health outcomes [28,29]. Evidence from supportive housing approaches shows that for people experiencing chronic homelessness and mental illness, housing-first interventions, via assertive community treatment or intensive case management, are more effective in terms of reduction in inpatient psychiatric hospital utilization than standard care provision [30,31]. All these aforementioned reasons support the notion that PEH need multidisciplinary and integrated primary healthcare management regarding somatic, mental, and social issues that aim at tackling barriers they experience [32,33]. Additional evidence supports a need for health interventions including elements of co-design in healthcare delivery design [26]. Traditionally, those affected by issues to be addressed with a given intervention are excluded from designing and decision-making processes, while mechanisms of inclusion and participation are suggested to yield greater long-term success and effectiveness in its implementation [30,31].

Given the global rise in the numbers of PEH and the need for evidence-based, effective interventions, this review aims to address this urgent need by summarizing the current evidence relating to mental health services in primary care for PEH. A preliminary literature search by the authors did not identify a systematic review that addresses experiences across mental health services in PEH with mental illness. In this comprehensive systematic review of data from quantitative studies, the authors aimed to synthesize the current evidence on the characteristics and effectiveness of co-designed mental health interventions in primary care for adult PEH with mental health illness and to compare models where the quality of the evidence permitted this. To provide a greater understanding of such interventions, this review can support policies and intervention strategies tackling mental healthcare in primary care settings for adult PEH.

## 2. Materials and Methods

This systematic review was conducted according to the Preferred Reporting Items for Systematic Reviews and Meta-Analyses (PRISMA) checklist and statement [34]. The protocol of this systematic review was registered in the PROSPERO database (CRD42022354017).

### 2.1. Search Strategy

**Figure 1:**
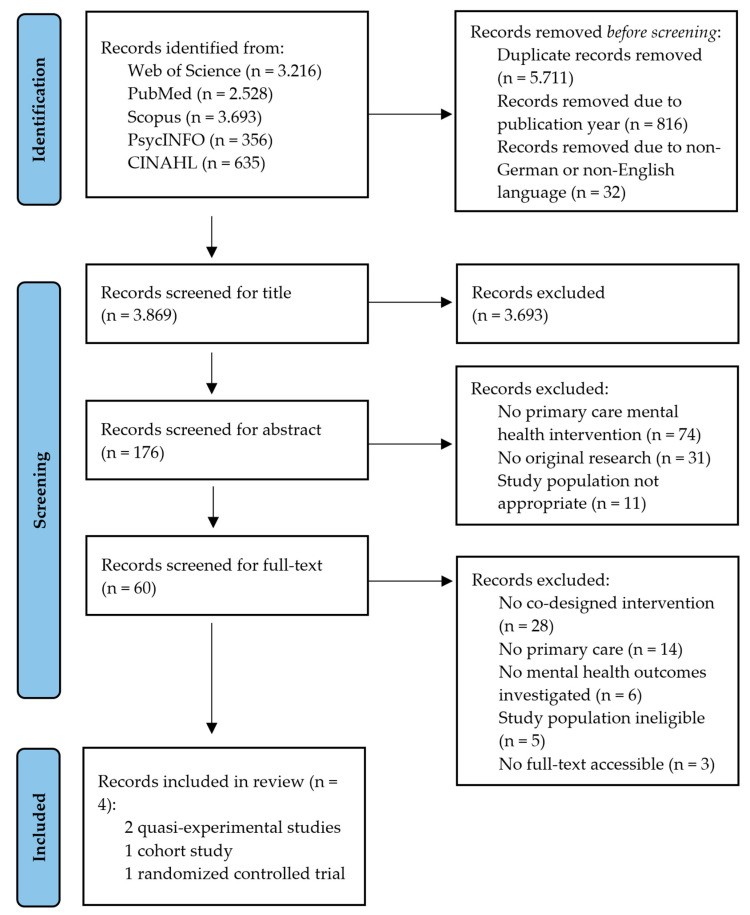
Flow chart of literature search.

Two of the authors searched papers from 1 January 2000 to 21 March 2022 in the electronic databases MEDLINE/PubMed, Scopus, Web of Science, PsycInfo, and CINAHL. In the search, only papers written in English or German were included, matching the authors’ language proficiency. To identify missing papers, bibliographies of all included studies were checked manually for additional references. The following search string was used across all databases: (homeless*) AND ((primary care*) OR (mental healthcare*) OR (collaborative care model*) OR (co-designed intervention*) OR (general pract*)) AND ((mental health*) OR (psych* diagnosis)). Figure 1 shows the entire search process in the form of a flow chart as suggested by PRISMA [35].

### 2.2. Criteria for Study Selection

This systematic review included cohort studies, intervention studies, case-control studies, cross-sectional studies, clinical trials, and randomized controlled trials (RCTs). Case reports, opinions, editorials, commentaries, letters, conference abstracts, and reviews or systematic reviews were excluded. Studies were included if they reported results from a co-designed intervention in a primary care setting in adult PEH, whereby homelessness was defined according to the categories of the European Typology of Homelessness and Housing Exclusion (ETHOS) [36], and interventions were identified as being co-designed if researchers included PEH and/or stakeholders in their model development collaboratively. However, other relevant interventions aiming at mental health improvements for PEH, such as collaborative care model-based interventions, integrated care interventions, shelter-based care interventions, as well as interventions that involved screening for diagnoses were also included. Additionally, multi-component interventions comprising management of physical health were included if they explicitly addressed mental health outcomes. Interventions can both be delivered by a multidisciplinary team of professionals or a single professional. Interventions with a multi-component approach that are partly delivered by healthcare professionals were also included. Among controlled study designs, we included studies that fulfilled both of our inclusion criteria for comparator or control groups: PEH aged 18 or above. Additionally, sites of implementation were assessed through the comparison of shelter or community-based services and interventions based in primary healthcare centers. Studies were excluded if participants in the study were children and minors or those who do not experience homelessness.

### 2.3. Types of Outcomes

For the review, we were interested in the relevant characteristics of co-designed interventions and their effectiveness in primary care settings aimed at improving the mental health outcomes of adult PEH. We defined effectiveness as compliance, rate of identified diagnoses, treatment rate, therapy outcomes, and satisfaction with the service. Additionally, we were also interested in implementation strategies known to impact the effectiveness of multidisciplinary collaborative care models aiming at mental health outcomes in PEH, as well as how the utilization of community-based primary care models or collaborative care models impacted mental health outcomes in PEH.

### 2.4. Data Extraction

While the title screening was performed by all reviewers, two independent reviewers initially reviewed all titles for duplication to ensure no relevant study was erroneously excluded, followed by two independent reviewers reviewing all abstracts. Subsequently, three reviewers independently assessed the full texts of all potentially eligible studies. If necessary, disagreements during the screenings of titles, abstracts, and full texts were resolved by discussion, involving another independent reviewer.

Guided by the data collection form provided by Cochrane Effective Practice and Organisation of Care [37], two independent reviewers (I.G. and T.S.) extracted the following information for all articles: (1) General information; (2) Eligibility; (3) Population and setting; (4) Methods; (5) Participants; (6) Intervention groups; (7) Outcomes; (8) Results; (9) Applicability; and (10) Other information.

### 2.5. Data Evaluation

The quality of the included studies was assessed by two reviewers using both the Tool to Assess Risk of Bias in Cohort Studies [38] and the Cochrane Risk of Bias Tool [39]. These tools allowed us to appraise the methodological quality of the three categories of studies included in our review, namely randomized controlled trials, non-randomized quasi-experimental studies, and cohort studies. Disagreements were resolved independently by a third reviewer.

## 3. Results

As represented in Figure 1, our initial search strategy yielded a total of 10,428 publications. After removing 6559 articles due to duplication, publication year, or language, 3869 records were identified as eligible for the subsequent screening. During the title and abstract screening process, a total of 3809 articles were excluded as they did not correspond to the review criteria. Out of 60 records examined in the final full-text screening, four studies fulfilled all inclusion criteria and were included for data extraction. Summarized methodological information on these studies can be found in Table 1.

Overall, most articles were excluded due to the following reasons: the investigated interventions were not set in primary care settings; the articles were not original research; the interventions were not created using a co-design approach; the study populations were not suitable; the studies did not investigate at least one mental health outcome.

Table 2 represents the quality appraisal of the included studies. The overall risk of bias in the randomized [40] and non-randomized [29,41] trials ranged from ‘unclear’ to ‘high risk’, while all articles were classified as ‘low risk’ in terms of the incompleteness of outcome data and selectivity in outcome reporting. In contrast, the cohort study conducted by Stergiopoulos et al. [42] showed ‘low risk’ throughout most domains of the used assessment tool.

### 3.1. Characteristics of Participants and Settings

Across all included studies, an aggregated number of 795 participants were investigated, while study-specific sample sizes varied from 67 to 363. In terms of gender distribution, three studies [40,41,42] included both male and female participants, and one study [29] focused on men exclusively as the research team collaborated with two shelters that served only men with complex mental health and social needs. Two of the studies were conducted in Toronto, Canada, one in Chicago, USA, and one in San Diego, USA. All of them investigated PEH who were experiencing impaired mental health, either by living with a mental illness [29,40,41] or unmet mental health needs as identified by healthcare providers [42]. Across all studies, the majority of participants living with a mental illness were experiencing anxiety disorders, bipolar affective disorders, major depression, posttraumatic stress disorders, schizophrenia or related psychotic disorders, or substance use disorder.

While Gilmer et al. [41], Stergiopoulos et al. [29], and Stergiopoulos et al. [42] did not focus on particular subgroups of PEH, Corrigan et al. [40] investigated people self-identifying as African American. They were reported as homeless if they did not have permanent housing and lived on the streets or in any other non-permanent or unstable situation, such as in a shelter, single-room occupancy, mission, vehicle, or abandoned building. Stergiopoulos et al. [42] conceived homelessness more narrowly since a person was only reported as being homeless if they were couch surfing, living on the street, or in a crisis or emergency shelter. While Stergiopoulos et al. [29] did not describe their definition of homelessness in detail, Gilmer et al. [41] explicitly focused on chronically homeless individuals. Individuals experiencing chronic homelessness were depicted as having spent several years living in precarious housing situations. The ETHOS category indicated in Table 1 was not reported in any of the studies presented here and was decided upon based on the population description in the individual studies.

Not all studies reported on inclusion and exclusion criteria for participation. While Gilmer et al. [41] did not state any eligibility criteria at all, Corrigan et al. [40] only excluded potential participants if they did not report currently having a mental illness, did not currently meet its definition for homelessness, or were already receiving case management services focusing on physical health at another institution. The two other studies clearly described both inclusion and exclusion criteria, whereby participants who were homeless and 18 years of age or above were included, and those who were a danger to themselves or others or had a history of severe aggression were excluded [29,42].

### 3.2. Characteristics of Interventions

Each of the included studies examined a different type of co-designed intervention, but they varied in terms of how they approached the co-design process. Corrigan et al. [40] investigated the peer navigator program that was developed by a team embedded in a community-based participatory research project. Based on existing peer navigator guidelines, findings from a previously conducted qualitative study, and team members’ individual experiences with mental and physical health systems, a patient navigator manual comprising several basic principles was created. During a treatment period of 12 months, peer navigators were supposed to approach program participants at least once a week in the form of a personal meeting. Service recipients were in charge of meeting locations and times. During these meetings, peer navigators reviewed the health concerns and worries of their clients and collaboratively established concrete actions to address these problems. For instance, the planned activities focused on homelessness, diet, or criminal justice involvement. Three participants who had experienced homelessness and recovered from severe mental illness served as peer navigators and shared their responsibilities for all assigned participants. The group of people within the peer navigator program was compared with a treatment-as-usual group utilizing a non-co-designed intervention.

During the intervention investigated by Gilmer et al. [41], PEH were provided subsidized permanent housing and team-based services focusing on recovery and rehabilitation. In these full-service partnerships, clients directly received services in their living environment including home, work, and other places they identified as favorable to themselves or where they needed support. Additionally, individuals were able to employ crisis intervention services operating 24/7. Utilizing this collaborative approach, clients were reportedly empowered to achieve their personal goals without this being determined by their respective mental illnesses. For study purposes, participants were contacted one year before engaging in the full-service partnership program while follow-up data collection occurred one year after their entry, resulting in a study period of two years. During this time, clients were not obliged to make use of therapy but had to meet with a treatment team at least once per month. Individuals were recruited from psychiatric hospitals, jails, shelters, emergency departments, county agencies, the street, and other localities or institutions. Community treatment teams were composed of several mental health and other professionals, as well as peer specialists providing their clients with various services such as medication management or substance abuse services. The intervention group was compared with users of unspecified public mental health services.

The integrated multidisciplinary collaborative care model studied by Stergiopoulos et al. [29] aimed at streamlining referrals, interdisciplinarity, increased provider communication, coordinated care, and integrated shelter-based case management. A team composed of shelter staff and (mental) healthcare professionals worked collaboratively within a treatment period of 12 months for each participant. During this period, PEH were interviewed at baseline, half-time, and end of the intervention. This model was compared with a shifted outpatient collaborative care model that aimed at providing PEH with timely mental healthcare, simplified referrals, and improved communication between shelter staff and local psychiatrists. As part of the model, the client’s treatment progress was appraised consistently as cared coordination and case management plans.

Stergiopoulos et al. [42] studied an interdisciplinary intervention providing PEH with case management, primary psychiatric care, peer support, and supplementary community services. The program called “coordinated access to care for the homeless” was a four-to-six-month intervention that included a variety of case management services, such as crisis intervention, community visits, or supportive therapies. Improved accessibility to support for issues surrounding income, health, and social issues was accomplished through collaboration with community health centers, social services, and other community agencies. This intervention used a combination of peer support and low-threshold healthcare. Healthcare professionals cooperated with case managers to create interdisciplinary assessments with PEH that allowed them to offer comprehensive and tailored care plans addressing clients’ needs. Since the study was designed as a cohort study, a comparison group was not needed.

### 3.3. Impact and Effectiveness of Interventions

As represented in Table 1, the included studies addressed several types of outcomes. Not all these outcomes were primarily focused on mental health. They included mental health (psychiatric disorder, mental health symptoms, and substance use; *n* = 3) [40,42], quality of life (general and disease-specific; *n* = 3) [40,41,42], service use (mental health services, general health services, and acute services; *n* = 3) [29,41,42], recovery (*n* = 2) [40,41], housing (housing and residential stability; *n* = 2) [29,42], physical illness (*n* = 1) [40], mental health services and housing costs on a system-level (*n* = 1) [41], community functioning (*n* = 1) [29], change in participant health status (*n* = 1) [42], and working-alliance construct (*n* = 1) [42]. These outcomes are described in more detail below, with a focus on the ones related to mental health and service use.

#### 3.3.1. Mental Health

Two studies directly investigated a total of three mental health outcomes, namely psychiatric disorders, mental health symptoms, and substance use. Psychiatric disorder, as measured by Corrigan et al. [40], was defined by the Texas Christian University Health Form (TCU-HF) and the 36-item Short Form of the Medical Outcomes Survey (SF-36). While the TCU-HF is based on a five-point Likert scale representing the sole frequency of 14 general health and ten mental health problems, the SF-36 considers an individual’s experience of health and several subfactors. Both the TCU-HF and the SF-36 demonstrated significant improvement in the psychiatric disorder outcome indicated by self-report indices, twelve months after baseline. Stergiopoulos et al. [42] examined the mental health state of their participants in terms of mental health symptoms as defined by the mental component score of the SF-36 and the 14-item Modified Colorado Symptom Index (CSI), and substance use, defined by a combination of drug and alcohol modules derived from the Addiction Severity Index (ASI), measuring substance use within the past 30 days. Six months after baseline, participants showed significant improvements in all scores, suggesting that interdisciplinary interventions may be promising in terms of improving mental health outcomes.

#### 3.3.2. Quality of Life

While two studies investigated the general quality of life of participants [40,41], one study focused on disease-specific quality of life [42]. The general quality of life was measured with Lehman’s Quality of Life Scale (QLS) by Corrigan et al. [40], whereas Gilmer et al. [41] compared quality of life cross-sectionally among clients receiving services in outpatient programs. Here, participants had to respond to 21 self-developed questions within eight domains. In both studies, participants in the intervention groups showed higher scores in the quality of life outcome. Stergiopoulos et al. [42] defined disease-specific quality of life as the total score of the 20-item Lehman Quality of Life Interview (QoLI-20), an instrument designed specifically for people living with severe mental illness. Analyzing the six-month versus baseline difference yielded a statistically significant improvement within the disease-specific quality of life outcome.

#### 3.3.3. Service Use

Besides outcomes related to mental health, the included studies also examined other variables that were not directly within the scope of our systematic review. Service use as an outcome was investigated in three of the included studies [29,41,42]. Notably, each study examined a different type of service. Gilmer et al. [41] analyzed the one-year standardized utilization of outpatient mental health services after participants had entered their intervention, and compared differences between individuals enrolled in a full-service partnership and those in a control group. They found an association between this program with significant increases in outpatient service use and days spent in housing. In addition, the researchers inquired into system-level costs for mental health services and housing and noted a reduction in inpatient and emergency service costs that compensated for 82% of the overall costs for full-service partnerships. Stergiopoulos et al. [29] did not specifically focus on mental health service utilization, but on health services in general. For that purpose, self-reported data on healthcare utilization were collected using several service use logs. Of interest were hospitalizations and emergency department visits in the six months prior, as well as visits to primary care physicians within the previous 30 days. In both investigated programs, a significant reduction in hospital and emergency services use was observed, while visits to community physicians increased. Stergiopoulos et al. [42] focused on acute service use, with self-reported numbers of hospitalizations and emergency department visits three months before follow-up data collection. Both numbers decreased significantly compared with the respective baseline data.

#### 3.3.4. Summary of Other Outcomes

Two studies also focused on recovery outcomes [40,41]. However, the definition of this outcome differed between those articles. Corrigan et al. [40] understood recovery as a more mental health-oriented variable that was measured using the Short Form of the Recovery Assessment Scale (RAS) including, e.g., willingness to ask for help and not being dominated by symptoms. The overall score of the RAS improved consistently over the course of the study, while participants in the treatment-as-usual group showed no change after 12 months compared with baseline. In turn, Gilmer et al. [41] approached the recovery outcome from the perspective of housing, financial support, and employment. The cohort of participants who received full-service partnerships had spent more days in housing, indicating a positive impact of the intervention. Furthermore, housing was investigated as an outcome in two other studies in terms of residential stability, whereby Stergiopoulos et al. [29] gathered self-reported residential data using residential logs from a database and Stergiopoulos et al. [42] used the Residential Time Line Follow-Back Calendar which captures housing history during the three months before a follow-up visit. Both studies yielded significant improvements in participants’ housing situations.

Physical health was assessed in two studies. Findings of both Corrigan et al. [40] and Stergiopoulos et al. [42] suggest a significant increase in participants’ physical health on the self-reported TCU-HF and the physical component score of the SF-36, respectively. Additional outcomes examined were community functioning in Stergiopoulos et al. [29] and the working-alliance construct described by Stergiopoulos et al. [42]. To assess community functioning, researchers used the self-rated version of the Multnomah Community Ability Scale that focuses on persons living with mental illness and their functioning within the community. Based on their findings, PEH experienced significant improvements in their community functioning. The working-alliance construct outcome was assessed using the Working Alliance Inventory-Participant which is a 12-item self-report instrument that measures the level of agreement between service providers and their clients about therapy goals, tasks, and the therapeutic relationship. Study findings after six months showed a significant and positive association between the inventory scores and the number of hospitalizations and emergency department visits.

## 4. Discussion

By conducting this systematic review, we sought to synthesize available evidence on co-designed interventions for mental health in primary care settings for people experiencing homelessness (PEH), focusing on program characteristics as well as impact and effectiveness. As far as we know, this is the first systematic review to focus on this specific topic.

The findings of our review revealed that using the co-design approach in terms of mental health promotion for PEH can yield significant benefits for this target population. Participants enrolled in the included studies consistently showed reduced symptoms of mental illness and substance use, indicating an improvement in terms of pre-existing psychiatric disorders. Moreover, other important spheres of life were positively affected, such as improved physical health or more stable living situations. In addition, the number of participants seeking acute healthcare services decreased, which was reflected in a lower frequency of hospitalizations or use of emergency care. Participants self-reported an increase in their quality of life, indicating targeted effectiveness. These positive findings are in line with other research conducted with underserved and marginalized populations, such as young people experiencing psychosis or culturally and linguistically diverse communities, and within the field of mental health service design demonstrating the advantage of the co-design approach for interventions targeting PEH and mental health [21,22,24,25,43].

Co-designed health and care interventions have been proven to be beneficial to PEH, specifically in the context of mental health promotion and care which can be confirmed by the findings of our review [44,45]. Particularly in this disadvantaged population, general health is consistently overshadowed by other serious problems of everyday life, resulting in higher levels of psychological distress leading to poor mental health [46,47]. This mental health vulnerability is further linked to a cascade of other social factors, such as lack of support or the inability to keep appointments, a vicious cycle that further compromises mental health [48]. In light of PEH frequently facing physical and mental health challenges together, this places an additional burden on individuals and further increases their level of vulnerability. Efficient primary care delivery aimed at promoting mental health, therefore, appears especially important [48,49,50], particularly in light of the beneficial effects of primary care on PEH as underscored in this review. By implementing primary healthcare, the development of severe mental health impairments can be counteracted, subsequently resulting in a positive impact on the overall social, health, and economic situation of PEH [50]. Adapting such primary healthcare interventions by implementing a co-design approach has the potential to make these even more effective.

### 4.1. Limitations

Despite promising findings, this systematic review is subject to some limitations. First, the generalizability of these results is not guaranteed as all included studies were conducted in North America and are therefore bound to a very specific social and healthcare setting. The number of included studies is considerably small, thus the generalizability of the results is not guaranteed. Since the four synthesized articles examined very different types of interventions, no mental health program was evaluated at least twice and independently. A comprehensive evaluation of the interventions, e.g., concerning their applicability in varying settings or with different subgroups of PEH is therefore impossible. The gender distribution in the reviewed studies prioritizes the experiences of male PEH. However, it should be noted that this imbalance might not reflect real-world data on the gender distribution of PEH as limited data regarding the gender-specific prevalence of homelessness exist [51]. Although research within the field of homelessness in European regions suggests a preponderance of male PEH, this gender distribution may be affected by European systems for enumerating homelessness that is undercounting women experiencing homelessness [52]. Women living homeless usually remain undetected for security reasons and because they make more use of private couch surfing arrangements than approaching public services. Additionally, when it comes to research concerning PEH and mental health, both researchers and policymakers may have misconceptions about their association [53].

A further limitation arises from the fact that of the included studies, only one was conducted using a randomized controlled design, which is considered the methodological gold standard within quantitative health research. The quality of quasi-experimental and cohort studies and the designs’ limitations in determining causal inferences received greater attention during risk of bias assessments. In general, a high to medium risk of bias was identified. The diversity of study designs also does not allow direct comparison, and especially complex real-life interventions can benefit from more experimental designs.

Another limitation is the ambiguity among the articles regarding the used definition of the co-design approach [19]. Specifically, none of the papers used the term co-design, which is why some uncertainties arose about study selection. For example, interventions were referred to as collaborative or developed in collaboration with peers but without using the term co-design. This inconsistency regarding the co-design approach led to the exclusion of numerous articles during the selection process and was resolved by constant and extensive discussion among the authors of this paper. We therefore also urge clearer definitions in the literature around the use and utility of the concept of co-design.

### 4.2. Recommendations

Selected recommendations for future research can be derived from this systematic review. Overall, the co-design approach provides an important tool with which to increase the efficiency and effectiveness of health interventions for PEH. Since mental health among PEH is a complex and challenging issue for health and care providers and decision-makers, more efficient approaches adapted to this population’s actual needs are required.

Results of our study show that to date, co-designed mental health approaches for PEH have hardly been used for the development of such programs even though deficits have long been identified [17,50], resulting in a lack of studies in this context. As co-designed interventions for other marginalized population groups have produced positive results in improved mental health, quality of life, housing, and acute and hospital service utilization [24], this gap is glaring. Moreover, due to the heterogeneity of the included studies, it remains unclear at what point PEH should be approached with co-designed primary mental health interventions for them to be most effective. Further research in this area is urgently needed to adequately provide PEH with the mental healthcare they need and empower them to create targeted interventions sensitive to their lived experiences.

## 5. Conclusions

In summary, co-designed mental health interventions represent an approach with great promise for benefitting PEH. Co-designing allows individuals who currently are or have been directly affected by homelessness to participate in the development process of a health promotion or prevention program alongside stakeholders and researchers. This approach can mean a considerable increase in quality and cost-effectiveness. In the context of PEH and mental health, co-designed programs have been insufficiently researched, yet the deployment of this approach can bring about sustainable improvement in numerous spheres of PEH’s lives. Given existing gaps in research, many aspects, for instance, regarding the components that such program interventions need for them to be successful, require further exploration to understand their effectiveness in varying settings and for varying subgroups of PEH.

## Figures and Tables

**Table 1:** Methodological information of included studies.

Authors	Sample Size	Study Design	Duration of Participation	Participants	ETHOS Category	Sex Distribution	Types of Intervention	Types of Outcome Measures	Results
Corrigan et al. [40]	67	RCT	12 months	African Americans with serious mental illness who were homeless	1.1	Male: 41 (61%); Female: 26 (39%)	A one-year trial of PNP compared with TAU	Physical illness; psychiatric disorder; recovery; quality of life	Significant improvement in self-reported physical and mental health, recovery, and quality of life for those in the PNP program compared with treatment as usual, while both groups improved their domicile and insurance coverage.
Gilmer et al. [41]	363	Quasi-experimental	24 months	Adult PEH with severe mental illness who were FSP clients and clients receiving public mental health services	1.1; 2.1	Male: 228 (63%); Female: 135 (37%)	Housing First programs that do “whatever it takes” to improve residential stability and mental health outcomes	Recovery outcomes; mental health service use; mental health services and housing costs from the perspective of the public mental health system; quality of life	Participation in an FSP was associated with substantial increases in outpatient services and days spent in housing. Reductions in costs of inpatient/emergency and justice system services offset 82% of the cost of the FSP.
Stergiopoulos et al. [29]	142	Quasi-experimental	12 months	Men experiencing homelessness and mental illness	1.1; 2.1; 8.1	Male: 142 (100%)	Shelter-based collaborative mental healthcare models IMCC and SOCC	Community functioning; residential stability; health service use	Participants experienced significant improvements in community functioning, housing, hospitalizations, emergency department visits, and community-based physician visits in both shelter-based collaborative mental healthcare programs over time. Due to the lack of observable differences, the less resource-intensive SOCC appears more favorable than the IMCC.
Stergiopoulos et al. [42]	223	Cohort study	6 months	Adult PEH with mental health needs	1.1; 2.1; 8.1	Male: 173 (78%); Female: 50 (22%)	CATCH program, a 4- to 6-month interdisciplinary intervention offering case management, peer support, access to primary psychiatric care, and supplementary community services	Change in participant health status; mental health symptoms; disease-specific quality of life; substance use; acute service use; housing; working-alliance construct	CATCH participants showed significant mental and physical health gain and reductions in mental health symptoms, substance use, and hospital admissions. An association was found between the strength of the participant-case manager working alliance and reduced healthcare use and mental health symptoms.

CATCH: coordinated access to care for the homeless; FSP: full-service partnerships; IMCC: integrated multidisciplinary collaborative care model; PEH: people experiencing homelessness; PNP: peer navigator program; SOCC: shifted outpatient collaborative care model; TAU: treatment as usual.

**Table 2:** Quality appraisal of included studies.

Authors	Quality Appraisal Tool	Domain	Risk of Bias
Corrigan et al. [40]	Risk of Bias assessment [37]	Random sequence generation	Unclear
		Allocation concealment	Unclear
		Blinding of participants and personnel	High risk
		Blinding of outcome assessment	Unclear
		Incomplete outcome data	Low risk
		Selective outcome reporting	Low risk
Gilmer et al. [41]	Risk of Bias assessment [37]	Random sequence generation	High risk
		Allocation concealment	High risk
		Blinding of participants and personnel	High risk
		Blinding of outcome assessment	Unclear
		Incomplete outcome data	Low risk
		Selective outcome reporting	Low risk
Stergiopoulos et al. [29]	Risk of Bias assessment [37]	Random sequence generation	High risk
		Allocation concealment	High risk
		Blinding of participants and personnel	High risk
		Blinding of outcome assessment	Unclear
		Incomplete outcome data	Low risk
		Selective outcome reporting	Low risk
Stergiopoulos et al. [42]	Tool to Assess Risk of Bias in Cohort Studies [38]	Selection of exposed and non-exposed cohorts was drawn from the same population	Low risk
		Confidence in the assessment of exposure	Low risk
		Confidence that the outcome of interest was not present at start of study	Low risk
		Matching of exposed and unexposed for all variables that are associated with the outcome of interest or adjustment of statistical analysis for these prognostic variables	Low risk
		Confidence in the assessment of the presence or absence of prognostic factors	Low risk
		Confidence in the assessment of outcome	High risk
		Adequacy of follow-up of cohorts	High risk
		Similarity of co-interventions between groups	High risk

## Data Availability

No new data were created or analyzed in this review. Data sharing does not apply to this article.
